# Enlargement rate of geographic atrophy before and after secondary CNV conversion with associated anti-VEGF treatment

**DOI:** 10.1186/s12886-020-01766-6

**Published:** 2021-01-05

**Authors:** Jakob Siedlecki, Caroline Koch, Benedikt Schworm, Raffael Liegl, Thomas Kreutzer, Karsten U. Kortuem, Ricarda Schumann, Siegfried G. Priglinger, Armin Wolf

**Affiliations:** Department of Ophthalmology, Ludwig-Maximilians-University, Mathildenstrasse 8, 80336 Munich, Germany

**Keywords:** Age related macular degeneration, Choroidal neovascularization, Geographic atrophy, Optical coherence tomography, Vascular endothelial growth factor

## Abstract

**Background:**

To study the enlargement rate of primary geographic atrophy (GA) before and after diagnosis of a secondary choroidal neovascularization (CNV) treated with anti-vascular endothelial growth factor (VEGF) therapy.

**Methods:**

Five hundred twenty-two consecutive eyes with primary GA were screened for the development of a complicating secondary CNV. Geographic atrophy was measured on blue autofluorescence (BAF) by two readers and calculated into mean growth rate before and after CNV diagnosis.

**Results:**

Ten eyes of six patients were included in the study (six study eyes with GA complicated by CNV, four GA only partner eyes). Follow-up was 1.42 ± 0.48 years before and 3.64 ± 2.73 years after CNV. There was no significant difference between mean growth rate before and after CNV (1.58 ± 0.99 vs. 1.39 ± 0.65 mm^2^/year; *p* = 0.44) or between study and partner eyes (*p* = 0.86). Over a mean time of 3.64 ± 2.73 years, a mean of 8.3 ± 2.8 anti-VEGF injections were given. No correlation between the amount of anti-VEGF injections and change in growth rate could be observed (r = 0.58; *p* = 0.23).

**Conclusion:**

In this pilot study, primary GA enlargement did not seem to be influenced by a secondary CNV. No association between the intensity of anti-VEGF treatment and changes in atrophy enlargement rates were found. Further studies with larger sample sizes are warranted.

## Background

Severe visual acuity loss is the natural course of late-stage AMD, defined by the development of choroidal neovascularization (CNV), or outer retinal and retinal pigment epithelium geographic atrophy (GA) [1]. Disease dynamics differ strongly between both late manifestations. The neovascular form exhibits a much more aggressive, and GA a markedly slower, but continuously progressing disruption of the retinal architecture, resulting in photoreceptor loss [1, 2]. While traditionally neovascular AMD and GA have been regarded as two different cul-de-sacs of drusen deposition, recent research indicates that both entities are much more closely intertwined. Five-year results of the CATT study have shown that 20% of patients with neovascular AMD under anti-vascular endothelial growth factor (VEGF) therapy develop GA after two years, doubling to 41% of patients at five years [3]. As a result, today severe vision loss under long-term anti-VEGF therapy is mainly caused by atrophic scars, which contrasts with pre-anti-VEGF era eyes mainly presenting fibrotic scars [3].

Many risk factors for the development of GA under anti-VEGF therapy have been identified, including partner-eye geographic atrophy [4, 5], suggesting that certain AMD phenotypes are especially susceptible to GA [6]. In contrast to these patient-specific risk factors currently not amenable to therapy, excessive anti-VEGF therapy as iatrogenic risk factor has been debated intensely after the 2012 CATT trial finding that patients treated with monthly anti-VEGF had a higher risk of GA as compared to pro-re-nata (PRN) dosing [5].

While almost all published works on the relationship of anti-VEGF and retinal atrophy enrolled patients with neovascular AMD, only two studies have investigated the effects of anti-VEGF in the case of primarily dry, atrophic AMD complicated consequently by CNV [7, 8]. Unlike the investigation of atrophy following neovascular AMD and anti-VEGF therapy, eyes with primary GA and secondary CNV offer the rare opportunity to compare GA enlargement rates before and after the diagnosis of a CNV, and thus study the impact of neovascularization and anti-VEGF therapy on GA. This study was therefore designed to compare GA enlargement rates before and after a secondary neovascular conversion, and to investigate possible associations with the resulting anti-VEGF therapy.

## Methods

### Participants

For this retrospective cohort study, the Smart Eye Database of Munich’s University Eye Hospital of Ludwig Maximilians-University, Germany, was screened for patients who were seen with age-related macular degeneration between 2007 and 2015. To study growth speed of GA before and after CNV diagnosis, inclusion criteria for this study were: 1. Diagnosis of AMD; 2. Diagnosis of geographic atrophy due to AMD without any signs of active or previous CNV on OCT and FAG/ICGA; 3. Lack of confounding comorbidities (diabetic retinopathy, hereditary retinal disease, diseases of the vitreoretinal interface, status after vitrectomy, optic media impeding sufficient image quality); 4. Follow-up > 2 years. As defined by Lois et al. [9], GA was defined as a reduced signal in both blue autofluorescence (BAF) and near-infrared reflectance (NIR) covering an area of > 0.05 mm^2^ without any confounding caused by hemorrhage, exudate, or blockage of the AF/NIR due to subretinal CNV. Institutional review board (University Eye Hospital Munich, Ludwig-Maximilians-University Munich) approval was obtained for this retrospective chart review. All patients provided informed consent prior to the first treatment, and the study adhered to the tenets of the Declaration of Helsinki.

Epidemiological data from all patients was gathered, including age, gender, ocular comorbidities, previous procedures, date of first diagnosis of GA due to AMD, date of first CNV diagnosis and anti-VEGF injection, number and type of anti-VEGF injections, and objective refraction-based visual acuity at baseline, date of CNV diagnosis, and end of follow-up using an ETDRS chart in a distance of 4 m.

### Multimodal imaging

Multimodal imaging was performed as needed at each visit after pupil dilation as described previously [10]. At each visit, spectral domain optical coherence tomography (SD-OCT) in volume mode (49 B-scans covering an area of 6 × 6 mm centered on the fovea), near-infrared (NIR)/blue autofluorescence (BAF) confocal laser scanning ophthalmoscopy (CSLO) were performed. and fluorescein (FAG) and indocyanine green (ICG) angiography at baseline, when CNV was suspected and when CNV activity was uncertain (all on Spectralis HRA + OCT, Heidelberg Engineering, Heidelberg, Germany).

Geographic atrophy size was documented on blue autofluorescence CSLO images obtained at 488 nm excitation (30° × 30°; 768 × 768 pixels; high speed mode). Quantification was performed using the area measurement tool in the Heidelberg Engineering proprietary analysis software (Heyex) by two experienced readers (JS and CK). The interclass correlation coefficient (ICC) between both readers was 0.98 (95% confidence interval: 0.97–0.99).

### Anti-VEGF treatment

CNV activity was defined on OCT as (I) any new macular fluid, (II) pigment epithelium detachment (PED) increasing central macular thickness > 50 μm, or (III) new or increasing macular hemorrhage. On FAG/ICGA, CNV activity was defined as exudation increasing with time. In case of newly diagnosed or reactivated CNV, intravitreal anti-VEGF injections were given using a pro-re-nata regimen. Substances used included ranibizumab (Novartis Pharma AG, Basel, Switzerland), aflibercept (Bayer Healthcare Pharmaceuticals, Berlin, Germany) and off-label bevacizumab (Genentech Inc., South San Francisco, CA, USA, and Hofmann-La Roche Ltd., Basel Switzerland). Treatment was stopped in the case of successful fluid elimination, and re-initiated when CNV reactivation was observed as defined above.

### Statistical analysis

Data were collected and analyzed in Microsoft Excel (Microsoft, Redmond, WA, USA). SPSS Statistics 23 (SPSS Inc., Chicago*,* IL*,* USA) was used for statistical analysis. The level to indicate statistical significance was defined as *p* < 0.05. The agreement of GA area measured by both readers was calculated as interclass correlation coefficient. The Shapiro-Wilk test was employed to test for normal distribution of the measured parameters within the study group. The Wilcoxon signed rank and the dependent samples t-test were employed to test for significant differences during follow-up within study and partner eyes. The Wilcoxon signed rank and the independent samples t-test were used for the comparison of study and partner eyes. Pearson’s correlation coefficient was used to test associations of dependent and independent variables. Graphs were plotted in Microsoft Excel showing the mean ± standard deviation.

## Results

### Baseline parameters

Out of 1632 eyes presenting with a dry atrophic macula between 2007 and 2015, 522 eyes with geographic atrophy (GA) due to age-related macular degeneration without significant other comorbidities were identified. After exclusion of patients with a follow-up < 2 years and the wrong sequence of events, i.e. GA secondary to neovascular AMD, six patients who presented with GA prior to CNV were identified. Out of these 12 eyes, two eyes of two patients suffered from submacular hemorrhage and fibrosis in the follow-up, which did not allow for sufficient imaging. Thus, 10 eyes of six patients were included in the study. There were six study eyes with the GA-CNV sequence and 4 partner eyes with sole GA acting as an intra-individual comparison group.

**Fig. 1:**
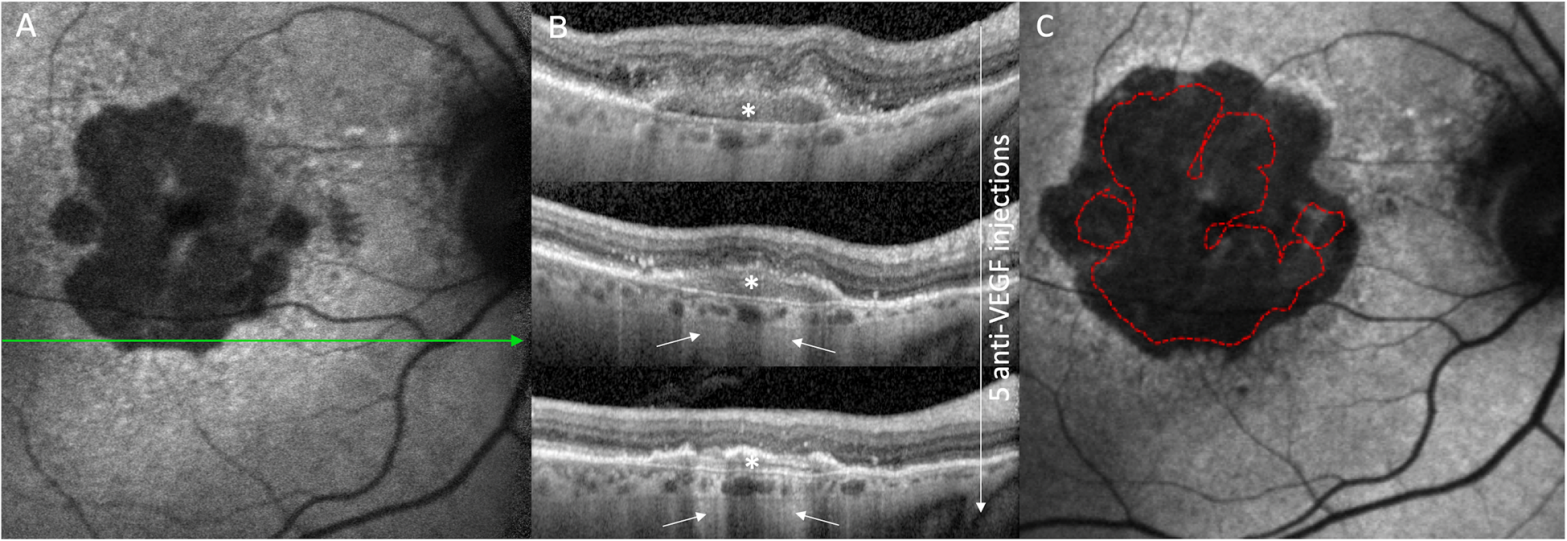
Patient example of primary geographic atrophy (GA) due to age-related macular degeneration complicated by the development of a choroidal neovascularization (CNV). Fourteen months after the baseline exam (**a**), a type 2 choroidal neovascularization (white *) was diagnosed on the lower border of the GA (**b**) and successfully treated with three monthly anti-VEGF injections. Due to reactivation, two further anti-VEGF injections were given, resulting in a morphologically partially fibrotic PED. On OCT, the areas of hypertransmission increased during follow-up. At the end of follow-up 21 months after the last anti-VEGF injection (**c**), the GA had considerably enlarged more in the three directions (nasally, temporally, superiorly) not affected by the CNV (lower aspect of GA)

Detailed baseline parameters of the study cohort can be found in Table 1. In brief, mean age of the six patients was 76.8 ± 2.0 (range: 72–79) years. Total follow-up was 5.06 ± 3.01 (2.01–9.68) years, split up into 1.42 ± 0.48 (0.77–1.80) years before and 3.64 ± 2.73 (1.15–7.88) years after CNV diagnosis. All eyes (100%) had subretinal drusenoid deposits (SDD) at baseline, while 5/10 (50%) also had soft macular drusen. All eyes (100%) showed hyperautofluorescence surrounding the GA area, of which 5 (50%) showed a banded, four (40%) a diffuse, and one (10%) a diffuse trickling phenotype as classified previously [11]. CNV were type 2 in all cases, with foveal involvement in 4 eyes (67%) and intraretinal fluid in all cases. A patient example can be found in Fig. 1.

**Table 1:** Patient demographic and baseline lesion data with GA enlargement characteristics

No. of eyes (n)	10
No. of patients (n)	6
Gender (m/f)	0/6
Mean age (y)	77 ± 2 (range: 75 to 79)
Mean follow-up (years)	5.06 ± 3.01 (2.01–9.68)
before CNV diagnosis	1.42 ± 0.48 (0.77–1.80)
after CNV diagnosis	3.64 ± 2.73 (1.15–7.88)
Mean BCVA (logMAR)	
Study eye (*n* = 6)	
baseline	0.84 ± 0.40 (0.30–1.30)
at CNV diagnosis	0.94 ± 0.44 (0.30–1.40)
end of follow-up	1.01 ± 0.57 (0.40–1.92)
Partner eye (*n* = 4)	
baseline	1.09 ± 0.56 (0.50–2.00)
end of follow-up	1.24 ± 0.48 (0.50–2.00)
Drusen	
Soft	5/10 (50%)
SDD	10/10 (100%)
Choroidal thickness	
Study eye	
baseline (μm)	152 ± 51 (71–224)
last follow-up (μm)	101 ± 44 (39–165)
Partner eye	
baseline (μm)	130 ± 63 (46–222)
last follow-up (μm)	127 ± 99 (23–290)
Geographic atrophy	
Study eye (n = 6)	
GA lesions baseline (n)	2 ± 1 (1–4)
Foveal involvement	4/6 (66.7%)
Phenotype	
Banded	3/6 (50%)
Diffuse	2/6 (33.3%)
Diffuse Trickling	1/6 (16.7%)
GA baseline (mm^2^)	2.11 ± 1.48 (0.79–4.35)
GA CNV diagnosis (mm^2^)	4.55 ± 2.98 (2.31–10.51)
GA last follow-up (mm^2^)	8.58 ± 3.50 (4.37–14.07)

### GA growth rate

**Fig. 2:**
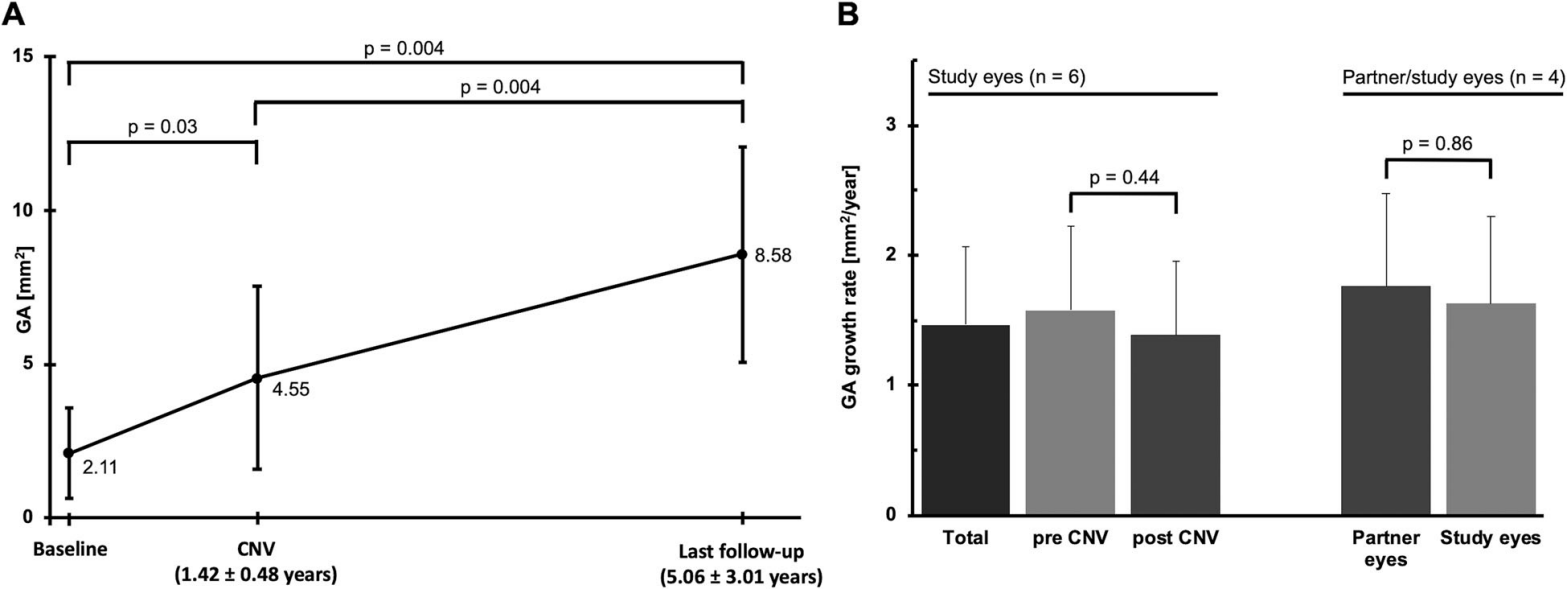
**a** Enlargement of GA before and after the diagnosis of a complicating CNV. The mean GA of 2.11 ± 1.48 (range: 0.79–4.35) mm^2^ at baseline increased to a mean 4.55 ± 2.98 (2.31–10.51) mm^2^ at CNV diagnosis after a mean 1.42 ± 0.48 (0.77–1.80) years (*p* = 0.03). In the following 5.06 ± 3.01 (1.15–7.88) years until end of follow-up, GA increased to a mean 8.58 ± 3.50 (range: 4.37–14.07) mm^2^ (*p* = 0.004), resulting in a total mean growth rate of 1.46 ± 0.82 (0.77–3.03) mm^2^/year (*p* = 0.004). **b** Rate of enlargement of GA before and after the diagnosis of a complicating CNV. There was no significant difference between the mean enlargement rate pre-CNV (1.58 ± 0.99) and post-CNV diagnosis with resulting anti-VEGF treatment (1.39 ± 0.65 mm^2^/year; *p* = 0.44). An intra-individual comparison of the total enlargement rate during the complete follow-up between study and partner eyes showed no significant difference (1.63 ± 0.99 vs. 1.76 ± 1.11 mm^2^/year; *p* = 0.86)

For the six study eyes, a GA at baseline of 2.11 ± 1.48 (0.79–4.35) mm^2^ increased significantly to 4.55 ± 2.98 (2.31–10.51) mm^2^ at CNV diagnosis (*p* = 0.03) and 8.58 ± 3.50 (4.37–14.07) mm^2^ (*p* = 0.004) at end of follow-up (Fig. 2a). Resulting total GA growth speed was 1.47 ± 0.82 (0.77–3.03) mm^2^/year. There was no significant difference between mean growth speed pre-CNV (1.58 ± 0.99 (0.68–3.46) mm^2^/year) and after CNV (1.39 ± 0.65 (0.80–2.49) mm^2^/year; *p* = 0.44). As classified by Holz et al. [11], three of the study eyes had a banded autofluorescence phenotype, two a diffuse and one a diffuse-trickling phenotype. The fastest total growth speed (up to 3.46 mm^2^/year) was exhibited by the only eye with a diffuse-trickling phenotype, while the slowest growth was observed in one of the eyes with a banded phenotype (0.68 mm^2^/year). Mean growth speed ratio for each eye (calculated as growth rate post-CNV/pre-CNV) was 0.97 ± 0.30 (0.59–1.40). All lesions diagnosed were type II CNVs with intraretinal fluid during follow-up. Foveal involvement was seen in 4/6 (67%) eyes. In two eyes (33.3%), the CNV was adjacent to the GA and slowed growth in this area was observed.

### Comparison of study and partner eye GA growth rate

**Fig. 3:**
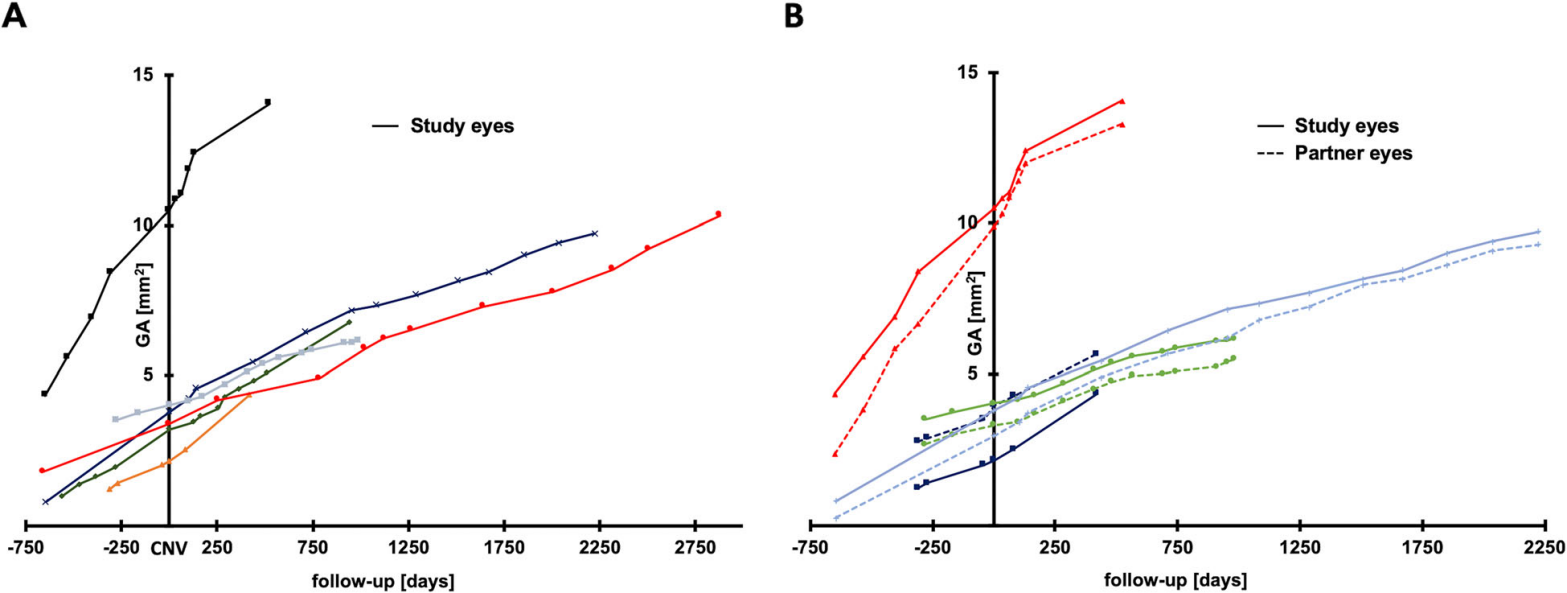
**a** Individual growth rates pre/post CNV for the six study eyes. Growth rate ratios ranged from 0.59 to 1.40, calculated as growth rate post/pre CNV. **b** Comparison of GA growth rates pre- and post-CNV between study eyes and partner eyes without signs of CNV (dotted lines). No significant difference in growth rate was observed (1.63 ± 0.99 (0.77–3.03) vs. 1.76 ± 1.11 (0.94–3.40) mm^2^/year; *p* = 0.86)

For the four partner eyes, which did not show any signs of active CNV during follow-up, mean GA area at baseline was 2.01 ± 1.19 (0.24–2.77) mm^2^, which was not different from the four corresponding study eyes (2.47 ± 1.73 (0.79–4.35) mm^2^). Enlarging to 8.74 ± 3.64 (5.67–13.29) mm^2^ at end of follow-up, the resulting total growth speed of partner eyes was 1.76 ± 1.11 (0.94–3.40) mm^2^/year, which was not statistically different (*p* = 0.86) from the four corresponding study eyes (1.63 ± 0.99 (0.77–3.03). A correlation of study and partner eye growth rates can be found in Fig. 3 B.

### Correlation of GA growth rate and anti-VEGF injections

Over the mean time of 3.64 ± 2.73 years with CNV diagnosis, a mean of 8.3 ± 2.8 (5–13) anti-VEGF injections were given. Of the 50 anti-VEGF injections in total, 27 were aflibercept (54%), 20 ranibizumab (40%) and 3 bevacizumab (6%). For 37 out of 50 injections (74%), new or increasing intraretinal fluid (IRF) associated with the CNV was the reason for treatment; the remaining 13 injections (26%) were performed due to new subretinal fluid.

**Fig. 4:**
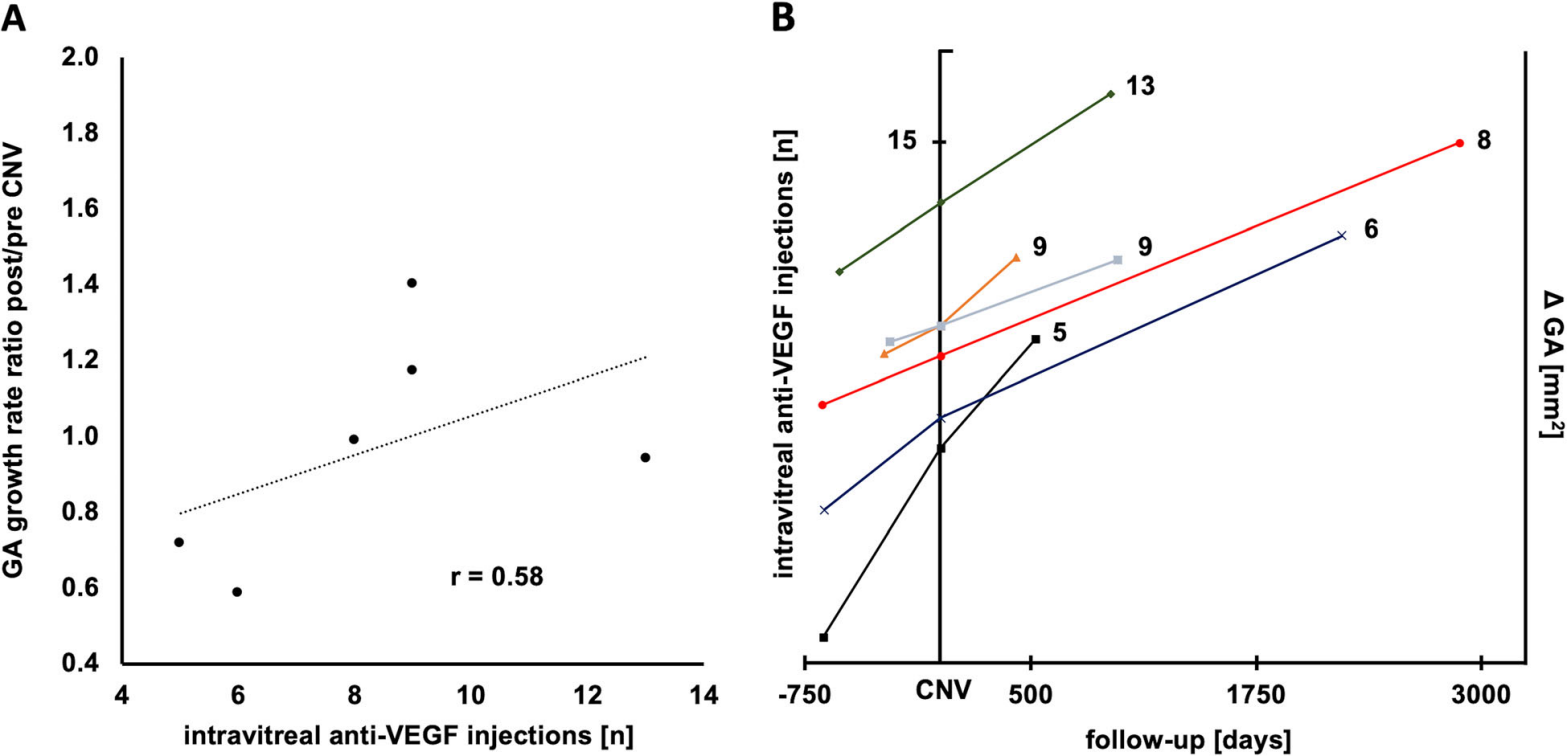
**a** Change in GA enlargement rate correlated with the total amount of anti-VEGF injections given. Change in enlargement speed was defined as the ratio of growth rate after CNV diagnosis until end of follow-up, divided by the growth rate prior to CNV diagnosis. Pearson correlation revealed no connection between changes in growth rate and the total or yearly amount of anti-VEGF injections given (total: r = 0.58; *p* = 0.23; yearly: r = 0.52; *p* = 0.30). Besides, no other investigated factor was found to be relevant (baseline GA size: r = − 0.06, *p* = 0.91; number of baseline GA lesions: r = 0.45, *p* = 0.38; subfoveal choroidal thickness: r = − 0.26, *p* = 0.62; age: r = − 0.04, *p* = 0.94; number of visits with IRF presence: r = − 0.44, *p* = 0.38). **b** Individual study eye growth rates pre- and post-CNV in mm^2^/year (x-axis and right y-axis) stratified by anti-VEGF injection number on the left y-axis. Higher cumulative anti-VEGF injection count did not correlate with growth rate acceleration

Associations of change in growth speed with baseline and treatment parameters can be found in Table 2. Pearson correlation showed no connection between post-CNV growth speed acceleration and the total amount of anti-VEGF injections given (r = 0.58; *p* = 0.23; Fig. 4a) or the amount of mean anti-VEGF injections per year (r = 0.52; *p* = 0.30). Besides, no other investigated factor was found to be relevant (baseline GA size: r = − 0.06, *p* = 0.91; number of baseline GA lesions: r = 0.45, *p* = 0.38; subfoveal choroidal thickness: r = − 0.26, *p* = 0.62; age: r = − 0.04, *p* = 0.94; number of visits with IRF presence: r = − 0.44, *p* = 0.38). Figure 4b shows the change in GA growth speed pre−/post-CNV for each eye stratified by anti-VEGF injection number.

**Table 2:** Correlation of baseline factors influencing change of growth speed after CNV diagnosis

Study Eye	GA growth speed (mm^**2**^/year) ratio pre post post/pre			Baseline GA (mm^**2**^)	Baseline GA lesions (n)	Baseline choroid (μm)	IRF presence (%)	Anti-VEGF IVOM (n)
**1**	1.70	0.97	0.59	0.79	1	71	0.21	6
**2**	3.46	2.49	0.72	4.35	1	154	0.22	5
**3**	0.89	0.88	0.99	1.79	4	127	0.44	8
**4**	0.68	0.80	1.17	3.52	1	165	0.36	9
**5**	1.28	1.79	1.40	1.22	3	172	0.93	9
**6**	0.85	1.65	0.94	1.20	3	224	0.31	13
**Pearson correlation coefficient (r) with change in growth speed of GA**				−0.06 *p* = 0.91	0.45 *p* = 0.38	−0.26 *p* = 0.62	−0.44 *p* = 0.38	0.58 *p* = 0.23

In the study eyes, visual acuity of 0.84 ± 0.40 (0.30–1.30) logMAR at baseline remained stable at CNV diagnosis (0.94 ± 0.44 (0.30–1.40); *p* = 0.39) and end of follow-up (1.01 ± 0.57 (0.40–1.92); *p* = 0.71).

## Discussion

Various risk factors for GA have been described, including SDD [12, 13], IRF [14, 15], reduced subfoveal choroidal thickness [14, 16] and age [17]. While these factors currently largely remain inaccessible to therapy, atrophy and atrophic scars can also result from CNV activity [18], which can be successfully managed by intravitreal anti-VEGF treatment. However, retrospective analyses of large clinical trials, including CATT [5] and IVAN [19], and smaller prospective studies [20] have suggested that excess anti-VEGF treatment, i.e. “drying the macula to excessively”, might increase the risk for GA and drive GA enlargement.

Almost exclusively all studies on the topic of GA and anti-VEGF have examined GA secondary to neovascular AMD. In contrast to studying nascent GA as a response to anti-VEGF, measuring the effect of anti-VEGF on a pre-existent GA offers the unique opportunity to validate a possible toxicity directly on well-established lesions with the corresponding pre-CNV growth rates acting as a valid intra-eye control group. In our study analyzing primary GA lesions that secondarily developed CNV and required anti-VEGF treatment, there was no significant difference between mean growth rate before CNV (1.58 ± 0.99 (0.68–3.46) mm^2^/year) and after CNV (1.39 ± 0.65 (0.80–2.49) mm^2^/year), resulting in a post/pre CNV enlargement ratio of 0.97. Moreover, there was no difference in enlargement rate between eyes developing CNV and their associated partner eyes. And strikingly, no correlation between the amount of anti-VEGF injections applied for CNV treatment and changes in GA enlargement rates were found, while the GA growth rate was 1.47 ± 0.82 (0.77–3.03) mm^2^/year, which is well in line with the literature reporting mean 0.53 to 2.6 mm^2^/year with a median of approximately 1.78 mm^2^/year [21].

These data suggest that exudative CNV development, if treated accordingly, and subsequent anti-VEGF treatment seem to represent secondary factors influencing GA with a much lesser impact than “genetically coded” risk factors, e.g. SDD, choroidal thinning, and age. Due to the presence of SDD in all eyes in our study, rather severe choriocapillary ischemia and thus stronger susceptibility for GA and faster enlargement rates could be assumed, with little secondary influence of a newly diagnosed exudative CNV.

In contrast to these data, a growing body of evidence suggests that type 1 CNV might protect overlying retinal areas from GA growth [22]. Due to the availability of OCT angiography, CNV presence can nowadays be frequently detected even in the absence of leakage, a finding which has been termed ‘quiescent CNV’ by many authors [23] and reproduces earlier histopathologic findings, which suggested subclinical CNV in up to 33% of eyes presenting with solely GA clinically [23]. Recently, de Oliveira Dias and colleagues found that most eyes with dry AMD developing exudation during follow-up already had quiescent non-exudative CNV at baseline [24]. In this scenario, the current aim of anti-VEGF therapy might be to restore and preserve an “anti-VEGF” equilibrium, in which exudative changes due to CNV are treated with repeat injections, and type 2 CNVs are pruned into mature type 1 lesions. Such trophic type 1 lesions can then persist in a non-exudative state and provide metabolic support to the RPE and retina, being tolerated as long as exudation is absent, and retreated with anti-VEGF when exudation and invasive growth recur.

As a limitation, our study does not provide OCT angiography imaging, which might have detected or excluded quiescent CNV at the time of inclusion. Moreover, a major limitation of our study certainly lies within its small sample size, which however results from the investigation of a rare, but very specific phenotypic presentation of AMD. Our study also lacked a defined follow-up schedule to have the possibility to begin intravitreal treatment promptly and thus improve comparability between eyes.

## Conclusion

In conclusion, our small pilot study suggests that primary GA enlargement rates are largely independent from complicating secondary CNV development and the resulting anti-VEGF therapy. In the ongoing debate on the relationship of anti-VEGF therapy and macular atrophy, this offers further reassurance to also perform anti-neovascular treatments in mainly atrophic lesion types. Due to the possibly protective effects of stable type 1 CNV, prevention of type 2 conversion and exudation might be the main therapy goal in this particular phenotype of AMD, while non-exudative type 1 lesions can be allowed to persist in a non-exudative trophic state. Further studies with a larger sample size are warranted.

## Data Availability

The datasets during and/or analysed during the current study available from the corresponding author on reasonable request.

## Abbreviations

AMD: Age-related macular degeneration
BAF: Blue autofluorescence
CNV: Choroidal neovascularization
CSLO: Confocal laser scanning ophthalmoscopy
FAG: Fluorescein angiography
GA: Geographic atrophy
ICC: Interclass correlation coefficient
ICGA: Indocyanine green angiography
IRF: Intraretinal fluid
MA: Macular atrophy
OCT: Optical coherence tomography
SDD: Subretinal drusenoid deposits
VEGF: Vascular endothelial growth factor
